# *In situ *lignocellulosic unlocking mechanism for carbohydrate hydrolysis in termites: crucial lignin modification

**DOI:** 10.1186/1754-6834-4-17

**Published:** 2011-06-14

**Authors:** Jing Ke, Dhrubojyoti D Laskar, Deepak Singh, Shulin Chen

**Affiliations:** 1 Department of Biological Systems Engineering, Washington State University, Pullman, Washington 99164-6120, USA

## Abstract

**Background:**

Termites are highly effective at degrading lignocelluloses, and thus can be used as a model for studying plant cell-wall degradation in biological systems. However, the process of lignin deconstruction and/or degradation in termites is still not well understood.

**Methods:**

We investigated the associated structural modification caused by termites in the lignin biomolecular assembly in softwood tissues crucial for cell-wall degradation. We conducted comparative studies on the termite-digested (i.e. termite feces) and native (control) softwood tissues with the aid of advanced analytical techniques: ^13^C crosspolarization magic angle spinning and nuclear magnetic resonance (CP-MAS-NMR) spectroscopy, flash pyrolysis with gas chromatography mass spectrometry (Py-GC/MS), and Py-GC-MS in the presence of tetramethylammonium hydroxide (Py-TMAH)-GC/MS.

**Results:**

The ^13^C CP/MAS NMR spectroscopic analysis revealed an increased level of guaiacyl-derived (G unit) polymeric framework in the termite-digested softwood (feces), while providing specific evidence of cellulose degradation. The Py-GC/MS data were in agreement with the ^13^C CP/MAS NMR spectroscopic studies, thus indicating dehydroxylation and modification of selective intermonomer side-chain linkages in the lignin in the termite feces. Moreover, Py-TMAH-GC/MS analysis showed significant differences in the product distribution between control and termite feces. This strongly suggests that the structural modification in lignin could be associated with the formation of additional condensed interunit linkages.

**Conclusion:**

Collectively, these data further establish: 1) that the major β-*O*-4' (β-aryl ether) was conserved, albeit with substructure degeneracy, and 2) that the nature of the resulting polymer in termite feces retained most of its original aromatic moieties (G unit-derived). Overall, these results provide insight into lignin-unlocking mechanisms for understanding plant cell-wall deconstruction, which could be useful in development of new enzymatic pretreatment processes mimicking the termite system for biochemical conversion of lignocellulosic biomass to fuels and chemicals.

## Background

Lignin is one of the structural components of the plant cell wall, and provides strength and rigidity in plant tissues [1]. It is highly resistant to enzymatic degradation because of its insolubility, chemical complexity and lack of hydrolysable linkages [2]. Softwood lignin is a polymer of high molecular mass, made up of two phenylpropanoid units derived from *p*-coumaryl and coniferyl alcohols. The phenyl moieties of these compounds are referred to as *p*-hydroxyphenyl (H) and guaiacyl (G) units, and they are linked together to form a complex three-dimensional structure that has proved difficult to characterize [3]. In general, characterization and compositional analysis for such biomacromolecules have been performed by chemically or thermally degrading the lignin into smaller monomeric derivatives, which are subsequently separated by means of chromatographic techniques [4].

In nature, cellulose and hemicelllulose, which comprise the major energy source in lignocellulosic biomass, are encrusted with lignin, which provides protection against enzymatic attack in lignocellulosic materials. However, wood-feeding termites (WFT) found in tropical savanna and forests are able to digest lignocellulosic substrates efficiently [2]. Therefore, it is likely that WFT have a lignin-preconditioning system that enables them to manage such efficient degradation of woody plants [2]. Several studies have reported on the degradation of wood by WFT. In our previous work, we demonstrated different oxygen concentrations and the lignin degradation/modification process degradation/modification process both occur in the gut in the whole gut passage of the lower termite, *Coptotermes formosanus *Shiraki [5,6]. Geib *et al. *[7] showed that there were significant levels of propyl side-chain oxidation (depolymerization), demethylation of the ring methoxyl group and ring hydroxylation of lignin after passage through the gut of a dampwood termite (*Zootermopsis angusticollis*). Likewise, Scharf and Tartar [8] suggested that marked lignin degradation should be the first step in the process of wood digestion in the gut of WFT. This statement has recently been supported by Tartar *et al. *[9] and Coy *et al. *[10] with the identification of lignin degrading/modifying gene candidates in the *Reticulitermes *gut. In all likelihood, the success of WFTs in wood-cellulose digestion is not only attributable to cellulases, but also to pretreatment factors that modify lignin and increase accessibility of wood cellulose. Hence, it sems likely that structural modification of lignin is crucial for deconstruction of the plant cell wall and utilization of the cellulose within it. However, how WFT overcome the lignin barrier and produce such enhanced accessibility to cellulose has not clearly been found yet.

In the present study, to further elucidate the lignin-unlocking mechanism in WFT, we fed *C. formosanus *Shiraki termites on Southern pine softwood, and analyzed the fecal materials using ^13^C crosspolarization magic angle spinning with nuclear magnetic resonance (CP/MAS NMR) spectroscopy, Py-GC/MS with internal standard, and Py-GC/MS in the presence of S in the presence of tetramethylammonium hydroxide (Py-TMAH-GC/MS), in order to understand the lignocellulosic structural change associated with the digestion process through the termite gut. Solid-state NMR can address chemical changes in the structure of lignocellulosic biomass, because it can provide spectra of whole wood and lignin without degradation or isolation of components [11,12]. Recently, our understanding of the diversity of structural modification in lignocellulosic biomass conversion has been advanced, in large measure due to the ability to explore such structures through ^13^C CP/MAS NMR spectroscopy [13]. Crosspolarization (CP) pulse sequences are intended to transfer magnetic polarization from abundant nuclei (1H) to rare nuclei (^13^C) (1.1% of the natural isotopic abundance), resulting in enhancement of the resonance signal from the rare nucleus [14]. This technique allows detection.] characteristic resonance chemical shift values, corresponding to individual cell-wall components of biomass such as cellulose, hemicellulose and lignin. We report a solid-state NMR study of control and WFT-digested softwood tissues (feces) using CP-MAS NMR.

Likewise, Py-GC/MS is also a widely used analytical tool to characterize recalcitrant macromolecules and polymeric samples at the molecular level [5,15,16]. Pyrolysis is designed to thermally degrade polymers into small fragments, which are then separated by gas chromatography and identified by MS [4]. The acquired pyrogram constitutes a 'fingerprint' of the starting macromolecule to give information about the relative amount of its monomeric components [17]. It is well established that analytical pyrolysis can be used to quantitatively assess the content of carbohydrates [18] and lignin [19] in wood, and the lignin composition [20,21] of the wood. Use of analytical pyrolysis to assess the lignin amount in Maritime pine (*Pinus pinaster*) and spruce wood samples, with a precision comparable with that of the reference Klason method, have also been reported [19]. Although there is plenty of information on the analytical pyrolysis of different wood types [22,23], reports on biologically modified wood are scarce. In this study, we employed the Py-GC/MS technique to characterize and quantify the monomeric composition of lignin, using an internal standard (3,5-dimethoxy phenol) added directly to the pyrolysis sample holder. With the correction factors calculated for the lignin-pyrolysis fragments, the method was used to determine absolute lignin amount in the softwood sample before and after termite degradation.

Although it is possible to obtain accurate quantification of monomeric lignin composition by Py-GC/MS, this technique is limited to analysis (by GC/MS) of polar pyrolyzates generated from nitrogenous material associated with the secondary reactions of pyrozylates during the pyrolysis process [24]. However, co-injection of a derivatizing chemical reagent (TMAH) during pyrolysis provides more flexibility to acquire structural information than in conventional pyrolysis as it protects thermolabile compounds and enables the chromatographic separation of both polar and non-polar targets in the same run, thus allowing the subsequent methylation of -COOH and -OH groups on lignin [15,16]. The combination of pyrolysis with *in situ *methylation using TMAH to depolymerize the fragments through methylation of methyl esters from carboxylic acids and methyl ethers from alcohols/phenols is an easy and efficient method for the characterization of lignin-derived compounds. Methylation of polar compounds formed from pyrolysis renders them more volatile and less polar, so that they may be analyzed more readily by GC, and pyrolytic reactions are accordingly minimized [25]. This method has proved to be a very useful technique for the characterization of polymers [26] and for *in situ *analysis of lignin in biomass [27]. Thus, to further elucidate the structural modification of lignin in softwood in response to digestion by termites, methylation by TMAH at 250°C for 30 minutes was used, which was later characterized by GC/MS analysis.

The analysis methods of ^13^C CP/MAS NMR, Py-GC/MS with internal standard, Py-TMAH-GC/MS require no solubilization, fractionation or isolation of components, rendering it possible to evaluate directly the compositional changes of the native structure of components such as lignin. This is different from one- or two-dimensional solution-state NMR spectroscopic structural analysis of lignin, for which isolation of lignin from the plant biomass is absolutely necessary. It is possible that during this lignin-extraction process, minor artificial structural modifications would be introduced into the lignin polymeric framework.

## Results and Discussion

### Solid-state NMR analysis

**Figure 1:**
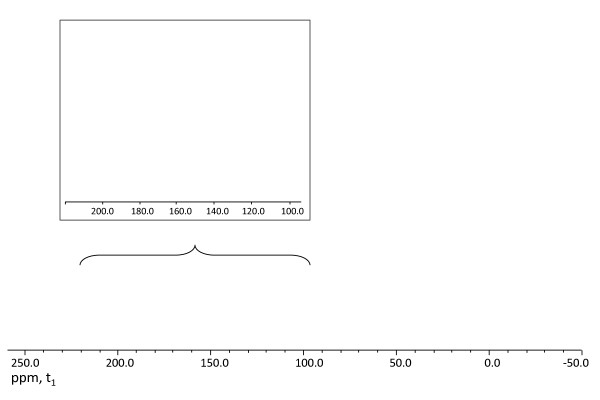
**Spectroscopy**. ^13^C 13C crosspolarization magic angle spinning and nuclear magnetic resonance (CP-MAS-NMR) analysis of the undigested control (red) and termite-digested softwood (black) samples. The inset represents the enlarged portion of the aromatic resonances.

Cell-wall tissues of control and termite-digested softwood tissues were subjected to ^13^C CP/MAS analysis under identical acquisition parameters. The resulting NMR spectra were processed with similar signal:noise ratio (Figure 1). A direct comparison of the overall pattern in the carbohydrate region (cellulose and hemicellulose region) of termite-digested and the softwood control, within an approximate range of chemical shift values of δ_c _= 109 to 60 ppm in the one-dimensional ^13^C CP/MAS NMR spectra, revealed a considerable difference in the intensities and distribution of the chemical resonances (Figure 1). Interestingly, a drastic decrease in the chemical resonance signal in the cellulose and hemicellulose region was documented in the termite-digested sample compared with the control. In the termite-digested sample, the resonance spectra at 75 and 73 ppm, corresponding to the C-2, C-3 and C-5 sugar carbon resonances [28] were relatively high, whereas the anomeric sugar C-1 and other C-4 and C-6 sugar carbon resonances, at 109, 90 and 65 ppm [29], respectively, were relatively diminished. This indicated the efficient degradation and/or hydrolysis of cellulose-derived moieties, as result of the cell-wall degradation by termites.

Attention was next directed to analysis of the aromatic region from 105 to 200 ppm in the ^13^C CP/MAS NMR spectra. The ascribed resonances in the aromatic region at δ_c _values of approximately 120, 140 and 150 ppm are assigned to lignin and lignin-derived product in both the spectra of control and termite-digested samples (feces). Accordingly, the signal at δ_c = _~120 ppm represents the unsubstituted aromatic carbon *ortho *and/or *para *to the substituted carbon, and the signal at δ_c = _~150 ppm corresponds to the substituted aromatic carbon of G units [13]. As the softwood lignin is mainly guaiacyl-derived, no resonance signal corresponding to syringyl (S) units (δ_c _= ~170 to 180 ppm) was seen in either termite feces or control softwood. Close inspection of the chemical resonance signal in the aromatic region (primarily at δ_c _= ~150 ppm) supported the existence of guaiacyl lignin-derived polymeric entities, in both the spectra of termite feces and control softwood but there was a significant divergence in the overall intensities between them. Termite feces had an increased level of the guaiacyl lignin-derived polymeric entities (Figure 1) with a dominant signal at δ_c _= ~150 ppm, which accounted for an increase of approximately 50% in the polymeric framework. This was also evident from the enhanced signal intensities at δ_c _= ~56 ppm, analogous to the methoxyl group (-OMe) of the aromatic ring of G unit, as seen for the termite feces compared with the undigested softwood control. Moreover, these results further indicated that digestion in the termite did not abruptly affect the dominant characteristics of the aromatic G units. These data suggest that during the lignocellulosic bioconversion process in the termite, no demethylation and/or demethoxylation reactions associated with the aromatic ring of the lignin itself occurred. Thus, the nature of resulting polymer in termite feces retained most of its original aromatic moieties after utilization of carbohydrate by the termite.

### Acetyl bromide analysis: lignin content analysis of control and termite-digested softwood

To quantitatively demonstrate the enrichment of the lignin-derived polymeric framework as result of the softwood-digestion process in termite, acetyl bromide (AcBr) analysis was performed on both the control and the termite-digested softwood tissues. AcBr analysis is a widely accepted method for estimation of lignin contents in various plant cell-wall residues (CWR) [30,31]. Therefore, extractive-free CWRs of both the control and termite-digested softwood tissues were individually treated with a reaction mixture consisting of 25% AcBr (v/v) in glacial acetic acid containing 4% perchloric acid, with the corresponding solubilized materials being individually measured by UV absorptivity (λ = 280 nm). An extinction coefficient of 20.09 l/g/cm [30,32] was used for estimation of lignin content. On application of the standard extinction coefficient to the AcBr analyses, the lignin content was found to be approximately 21.2% (212 mg/g of CWR) and 58.9% (589 mg/g of CWR) for the control and termite-digested softwood tissues, respectively. We should mention here that, because of the possibility of contamination by minor amounts of undigested wood powder in the termite feces, the lignin content as determined in the termite feces could be slightly underestimated. These results strongly indicated increased accumulation (~2.7 times) of lignin-derived entities in the termite-digested tissues. This is only possible if efficient degradation and utilization of cellulose and hemicellulose occurred to a significant scale compared with lignin during the softwood-digestion process, resulting in increased depostion of the lignin-derived polymeric framework in the termite feces. These results strongly support the results of the solid-state ^13^C CP/MAS NMR analysis, which also indicated an increased level of the lignin-derived polymeric entities in the termite feces.

### Absolute quantification of pyrolysis products from termite feces

**Figure 2:**
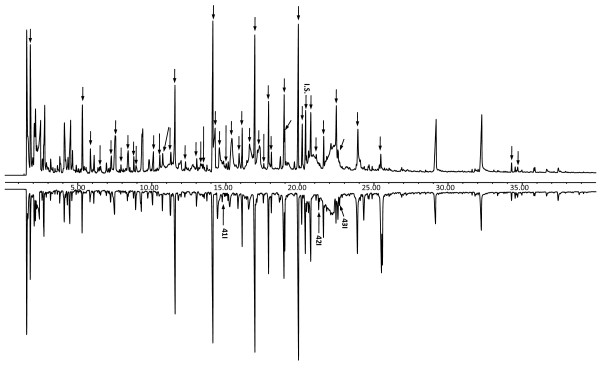
**Pyrogram of (A) **undigested softwood and (B) termite feces **(1 mg) with added internal standard (3,5-dimethoxyphenol, 50 μg)**. Numbers areas in Table 1. IS = internal standard. The peaks labeled from 41I to 43I are novel compounds that appeared in the fecal sample after digestion by termites. The new peaks and the IS are labeled by arrows and numbers; all the other phenolics and levoglucosan are labeled by arrows only. The structures of the labeled compounds are shown in Figure 3.

**Figure 3:**
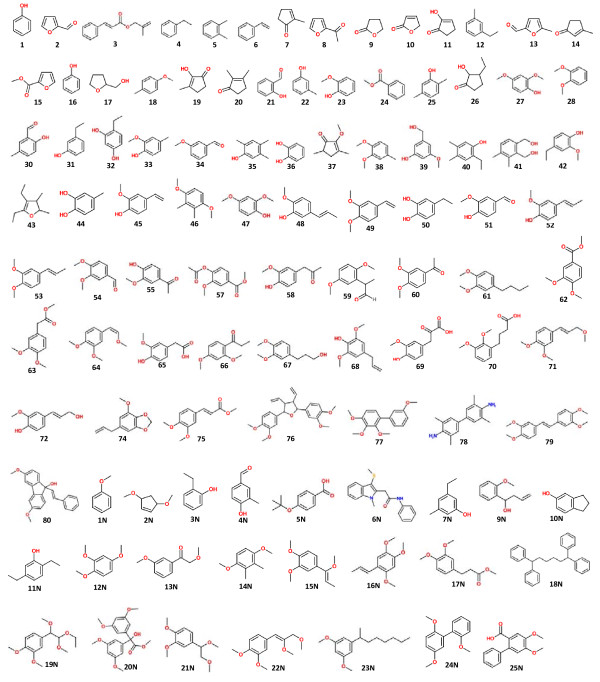
**Compound structures**. Assignment of all the structures of compounds labeled in Figure 2.

Figure 2 shows the total ion chromatogram (TIC) of each sample (1 mg) with addition of internal standard (0.05 mg). The corresponding pyrolyzed lignin-derived products with their absolute amounts (mg/g, mean value of three analyses), calculated using the internal standard method are also shown (Table 1; Figure 3).

**Table 1:** Pyrolysis products of each sample

Number	RT^b^, minutes	Compound identification^c^	Content of compound, mg/g		
				
			Undigested wood	Feces	Change, %
**1I**	1.811	1-Propen-2-ol, acetate	10.75 ± 0.51	4.74 ± 0.23	-55.9 ± 2.7

**2I**	5.319	Furfural	5.47 ± 0.26	2.27 ± 0.11	-58.6 ± 2.8

**3I**	5.866	2-Furanmethanol	2.01 ± 0.09	1.02 ± 0.04	-49.2 ± 2.1

**4I**	6.547	2-Cyclopentene-1,4-dione	1.70 ± 0.08	0.60 ± 0.03	-64.5 ± 3.1

**5I**	7.274	2(5H)-Furanone	2.62 ± 0.12	1.34 ± 0.06	-48.7 ± 2.2

**6I**	7.533	1,2-Cyclopentanedione	6.58 ± 0.32	2.81 ± 0.13	-57.3 ± 2.8

**7I**	7.926	Dihydro-3-methylene-2,5-furandione	1.17 ± 0.04	0.43 ± 0.02	-62.6 ± 2.4

**8I**	8.409	3-Butyldihydro-2(3H)-furanone	2.97 ± 0.10	1.21 ± 0.04	-59.2 ± 2.0

**9I**	8.780	Resorcinol	1.33 ± 0.06	0.58 ± 0.03	-56.2 ± 2.7

**10I**	8.940	Phenol	1.04 ± 0.05	1.47 ± 0.07	+42.6 ± 2.0

**11I**	10.109	3-Methyl-1,2-cyclopentanedione	2.67 ± 0.11	1.16 ± 0.05	-56.6 ± 2.3

**12I**	10.553	4-Methyl-5H-furan-2-one	2.07 ± 0.09	N/A	-

**13I**	10.739/11.263	3-Methylphenol	9.98 ± 0.47	3.99 ± 0.19	-60.0 ± 2.9

**14I**	11.567	2-Methoxyphenol	8.65 ± 0.41	8.64 ± 0.41	-0.1 ± 0.0

**15I**	12.283	Maltol	1.36 ± 0.06	N/A	-

**16I**	13.038	2,5-Dimethylphenol	1.38 ± 0.06	1.33 ± 0.06	-3.7 ± 0.2

**17I**	13.314	5-heptyldihydro-2(3H)-Furanone	1.79 ± 0.08	N/A	-

**18I**	13.469	2,3-Dihydroxybenzaldehyde	1.22 ± 0.05	N/A	-

**19I**	13.724/14.125	2-Methoxy-4-methylphenol	12.27 ± 0.42	12.51 ± 0.48	+2.0 ± 0.1

**20I**	14.280	4-Methoxy-2,5-dimethyl-3(2H)-furanone	7.33 ± 0.25	N/A	-

**21I**	14.569	1,2-Benzenediol	7.45 ± 0.35	4.08 ± 0.19	-45.2 ± 2.1

**22I**	15.027, 19.643, 20.902, 17.140-17.257, 21.101, 21.817, 22.102-22.500	*O*-D-Glucopyranosyl-D-glucopyranoside	34.14 ± 1.65	13.79 ± 0.67	-59.6 ± 2.9

**23I**	15.415	5-(Hydroxymethyl)-2-furancarboxaldehyde	8.80 ± 0.33	N/A	-

**24I**	15.898	3-Methyl-1,2-benzenediol	2.33 ± 0.08	1.974 ± 0.06	-15.2 ± 0.5

**25I**	16.101	4-Ethyl-2-methoxyphenol,	3.28 ± 0.16	3.194 ± 0.15	-2.7 ± 0.1

**26I**	16.601	4-Methyl-1,2-benzenediol	8.33 ± 0.39	3.337 ± 0.16	-59.9 ± 2.8

**27I**	16.955	2-Methoxy-4-vinylphenol	11.29 ± 0.46	14.246 ± 0.57	+26.2 ± 1.0

**28I**	17.563	4-(2-Propenyl)phenol	0.97 ± 0.05	1.383 ± 0.06	+42.4 ± 2.0

**29I**	17.887	Eugenol	4.85 ± 0.23	5.336 ± 0.25	+10.0 ± 0.5

**30I**	18.072, 20.156	2-Methoxy-4-propylphenol	5.20 ± 0.25	3.878 ± 0.18	-25.5 ± 1.2

**31I**	18.935	Vanillin	7.67 ± 0.33	10.594 ± 0.46	+38.2 ± 1.7

**32I**	19.901	Isoeugenol	12.03 ± 0.44	14.113 ± 0.05	+17.3 ± 0.8

**33I**	20.730	1-(4-Hydroxy-3-methoxyphenyl)-ethanone	8.38 ± 0.32	6.966 ± 0.62	-16.8 ± 0.8

**34I**	21.618	1-(4-Hydroxy-3-methoxyphenyl)-2-propanone	1.95 ± 0.04	5.984 ± 0.26	+207.1 ± 0.4

**35I**	22.447/24.315/25.574	4-((1E)-3-Hydroxy-1-propenyl)-2-methoxyphenol	8.13 ± 0.31	11.385 ± 0.33	+39.9 ± 0.8

**36I**	22.563	4-Hydroxy-3-methoxy-benzoic acid	2.72 ± 0.09	2.498 ± 0.29	-8.2 ± 0.3

**37I**	23.905	4-Hydroxy-3-methoxy-benzeneacetic acid	7.43 ± 0.31	7.508 ± 0.44	+1.0 ± 0.0

**38I**	25.458	4-Hydroxy-2-methoxycinnamaldehyde	1.75 ± 0.08	7.515 ± 0.28	+328.9 ± 2.3

**39I**	34.297	10,11-Dihydro-10-hydroxy-2,3-dimethoxydibenz	0.58 ± 0.02	0.706 ± 0.30	+22.2 ± 1.0

**40I**	34.703	Dihydrofisetin	0.81 ± 0.04	0.611 ± 0.29	-24.6 ± 0.0

**41I**	14.768	2,3-Dihydro-benzofuran	N/A	1.150 ± 0.03	-

**42I**	21.265	Benzoic acid, 4-hydroxy-3-methoxy- methyl ester	N/A	1.946 ± 0.09	-

**43I**	22.671	2,4'-Dihydroxy-3'-methoxyacetophenone	N/A	1.330 ± 0.04	-

^a^Identified products from thermal degradation of lignin and their absolute amounts (g/g, mean of three replicate analyses) calculated using the correction factor value.

^b^Retention time.

cSee the structures of the labeled compounds in Figure 5.

As evident from ^13^C CP/MAS spectroscopic studies, the lignin content in termite feces was concentrated compared with the undigested wood, revealing the relative amount of lignin-derived components increased in the termite feces. Py-GC/MS analysis displayed some new pyrolyzed compounds from termite feces (compounds 41I, 42I, and 43I in the spectra); indicative of possible oxidation on the side chain of lignin itself. Indeed, some pyrolyzed aromatic compounds largely increased after digestion by termites, such as compounds 34I and 38I, whereas the amounts of compounds with di-substituted hydroxyl groups on the aromatic ring decreased in the fecal samples (compounds 18I, 21I, 24I and 26I), signifying a possible dehydroxylation reaction occurring on the ring of the lignin G units, as a result of the termite-digestion process. The most abundant compounds found in the pyrograms of both the undigested control sample and of the feces were 19I (122.662 ± 4.178 mg/g) and 32I (141.131 ± 0.502 mg/g). The satructures were similar in both samples, being mainly composed of G-derived units; however, structural modifications were identified in their side chain-derived components. Some other important pyrolysis fragments were compounds 14I, 17I, 29I and 31I. These phenolics are the pyrolyzates from thermal cleavage at different sites of the phenylpropanoid structure of lignin, and show characteristics of the lignocellulosic material [33]. However, the changes in their relative amounts (Table 1) represented a plausible termite-induced modification of the lignin structure, leading to pyrolytic cleavage at diverse sites in the undigested and fecal samples.

Previously, Bocchini *et al. *[4] reported the total amount of phenolic compounds, related to the amount of lignin present in the softwood, to be 23.5 g/g, but in lignocellulosic samples, the pyrolysis yield for core lignin was less than 20% of the reported amount [34]. Based on a pyrolysis yield of 20%, the total amounts of lignin thermal-degradation products in the undigested and termite-digested softwood material, as obtained using the internal standard method, were 0.16 mg/g and 0.152 mg/g, respectively. These values are consistent with available data obtained using more 'classic' analytical techniques, such as neutral-detergent fiber analysis [35] or Klason lignin determination [18,36], which were estimated to be in the range of 0.1 to 0.2 mg/g. Furthermore, it is interesting to note here that, under identical pyrolysis conditions, the overall yield of released thermal lignin-degradation products in the feces (0.152 mg/g) was slightly reduced, although the fecal material was confirmed by^13^C CP/MAS analysis to contain a higher amount of lignin-derived polymer. Such an observation supports the possible structural modification within the lignin polymeric framework (for example,, as a result of dehydroxylation). Our previous data from three-stage pyrolysis GC/MS analysis and thermogravimetric (TG) analysis indicated that there was more lignin-derived residue preserved in termite feces after pyrolysis even after a temperature of > 600°C. This can be explained by the presence of aromatic modifications in the lignin itself during the digestion process in termites, which could result in the formation of a more condensed and stable structure that would require greater thermal energy for thermolysis and/or thermal degradation [37]. These results closely correlate with our recent findings for the TG analysis of termite feces, which found that a higher energy of activation was needed for the lignin region compared with the control, thereby indicating the requirement for higher thermal energy to produce oxidation of such structurally modified lignin.

### Py-TMAH-GC/MS analysis for lignin modification by termite

**Figure 4:**
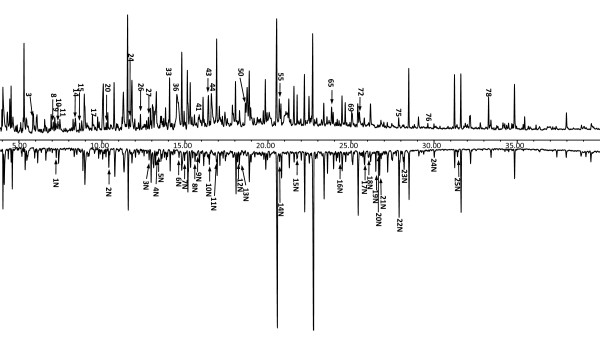
**Pyrogram**. Flash pyrolysis with gas chromatography mass spectrometry in the presence of tetramethylammonium hydroxide(Py-TMAH)-GC/MS profiles of **(A) **undigested softwood and **(B) **termite feces. (The peaks labeled as 1N to 25N in the fecal spectrum are novel pyrolyzed compounds that appeared after digestion by termites. All the peaks indicated in the undigested control spectrum are pyrolyzed compounds that were not present in the fecal sample. he structures of the labeled compounds are shown in Figure 5.

**Figure 5:**
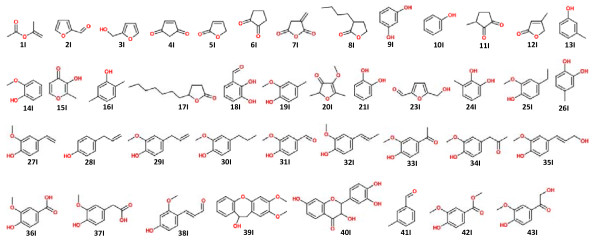
**Compound structures**. Assignment of all the structures of compounds labeled in Figure 4 and Table 1.

The TICs obtained from the pyrolysis and methylation of the initial control softwood and feces are depicted in Figures 4 and 5. The relative proportions of hemicellulose/cellulose-derived and lignin-derived products reflect the composition of cellulose, hemicelluloses and lignin components of both the control and termite feces. However, the small area assigned to cellulose and hemicellulose confirms the low efficacy of thermochemolysis in detecting carbohydrate units in polysaccharides [38]. In the pyrogram of the undigested sample (Figure 4, compounds 2, 7-11, 13-15, 17, 19, 20, 26, 37, 43; downward arrow), originating from pyrolysis of xylan and cellulose-derived moieties, pyrolyzed monomer sugars were detected mostly as unmethylated derivatives. This is possibly a result of the presence of the lignocellulosic matrix, with lignin as a protective barrier between the hemicellulose and cellulose polymeric framework, thereby preventing chemical access to this framework by the derivatizing agent (TMAH), and then making methylation difficult during the pyrolysis of the undigested softwood tissues. By contrast, the termite feces displayed relatively lower amounts of pyrolyzed products derived from xylan and cellulose moieties, and when they were present, they were in the methylated form. This might be the result of the degradation process of cellulose and hemicellulose of the softwood by the termite, thus allowing more of the remaining (unutilized) cellulose and xylan-derived polymeric framework in the feces to be chemically accessible to TMAH derivatization, allowing formation of pyrolyzed methylated compounds such as 37 and 2N.

It should be noted that gymnosperm lignin is known to be primarily composed of monomethoxyyphenol units (guaiacyl) [22]. The Py-GC/MS analysis of softwood tissues resulted in intense GC signals, which were primarily derivatives of guaiacyl structures (Figure 4; Table 1). Similarly, Py-TMAH-GC/MS analysis of control softwood also produced abundant peaks, rich in the dimethoxy-substituted benzenoid compounds. These peaks arose from G-lignin, while the major substructural attributes of the β-aryl ether subunits were retained. The reason for this is that Py-TMAH effectively cleaves the β-aryl ether subunits and by subsequent methylation, renders the released fragments detectable to GC/MS analysis [39,40]. In addition, the selective methylation on the lignin-derived pyrolytic compounds could also be explained by steric factors influencing the reaction between TMAH and phenolic hydroxyl groups [41].

To confirm the nature of the product profile of the fecal samples as obtained by Py-TMAH-GC/MS analysis, the major compounds resulting from TMAH derivatization and subsequent thermolysis were compared with those of the control. Thus, the quantitative differences (represented by peak height ratios) between the relative yields of pyrolyzed product were considered to be indicative of differences in the lignin structure of the original wood and termite feces. The product profile of the fecal sample was qualitatively similar to that of the control except for the presence of some new pyrolyzed products. The major compound in the pyrogram of control sample was identified as compound 54, whereas the most intense peak in the fecal sample, eluting at 22.69 minutes, was compound 62. Both these compounds represent oxidized products of polymeric G-derived lignin, which differ only in their side chain, as a result of lignin modification by the termites. This result indicated the generation of a lignin-derived polymeric entity in the termite feces, which retained the identity of the original aromatic moieties (G unit). Moreover, the occurrence of the derived compounds 19N and 21N in the pyrogram of the fecal sample represents the regiochemistry of the *β*-*O*-4' (*β*-aryl ether)-linked phenylglycerol-containing subunits in lignin, as reported from the selective labeling studies previously presented by Filley *et al *[40].

Furthermore, the cinnamyl alcohol and aldehyde end groups are also important for the evaluation of the total lignin structure because they can serve as a sensitive index for the structural changes and the overall character of the lignin [42]. Hence, the disappearance of compound 72, the cinnamyl alcohol end group, which was present in the undigested control but not in the fecal sample, indicates lignin modification at the substructural and/or interunit level by the digestion by termites. Some dimeric aromatic structures (compounds 76 and 79) were detected in the undigested softwood, as TMAH thermochemolysis preserves derived pyrolyzed products from the lignin *β*-5 and *β*-*β *substructure [24]. However, these structures were not present in the pyrolyzed fecal sample, indicating modification of these substructures as a consequence of digestion by termites.

In addition, production of new, possibly lignin-derived, pyrolyzed products (for example, compound 1N instead of 25N; Figure 4) was also evident in the Py-TMAH-GC/MS profile of the termite feces, in which 22N was the most abundant product, although other compounds including 11N, 21N and 23N, were also present in relatively large amounts. The origin of the pyrolyzed products 21N and 22N could be attributed to the pyrolytic dehydrogenation product of the β-aryl ether G-derived lignin substructure and/or interunit linkages, indicating conservation of the major *β*-*O*-4' interunit linkage even after digestion by termites. By contrast, formation of 11N and 24N suggested possible condensation of the structure of the resulting lignin-derived polymer in the termite feces, even though the abundant *β*-*O*-4' interunit linkage and most of its original aromatic residues were retained, wand were probably interconnected by additional interunit linkages. Overall, Py-GC/MS in the presence of TMAH generated more new pyrolyzates, indicating that the pyrolysis-methylation analysis with TMAH helped to preserve more of the information relating to the original lignin structure. Because TMAH depolymerizes the lignin molecule into its aromatic subunits by specifically cleaving the *β*-O-4 linkages and methylating all ring hydroxyls, it is different from the non-selective pyrolysis cleavage [43]. On an absolute level, the changes in the lignin structure are minor, which is in accordance with our previous hypothesis that there is not much degradation of lignin itself, but rather modification of specific lignin functional groups and linkages to assist in the unlocking of the carbohydrate.

Notably, our evidence from both solid-state ^13^C CP/MAS NMR and Py (TMAH) -GC/MS did not provide support for the existence of demethylation reactions associated with the aromatic ring of the lignin itself, which has been reported in earlier studies on lignin degradation by termites [7]. Further studies (using solution-state, long-range, two-dimensional heteronuclear multiple bond coherence in combination with heteronuclear multiple quantum coherence-total correlation spectroscopy and NMR) on lignin isolates from both termite feces and undigested control samples will be carried out in the near future to establish and confirm the reaction and bond-cleavage pattern and the subsequent compositional changes in the interunit linkages (substructures) of the lignin primary structure(s).

In this study, the ^13^C CP/MAS NMR spectroscopic analysis provided clear evidence that the aromatic polymer remaining after extensive cell-wall degradation in termite was still recognizable as a lignin-derived polymer. The Py-GC/MS method with internal standard allowed the absolute quantification of the pyrolysis products of the resulting lignin-derived polymer, and indicated that the main modification caused by the termites was dehydroxylation and structural modification in the intermonomer side-chain linkages of the native lignin. In addition, the Py-TMAH-GC/MS studies substantially suggest additional condensed interunit linkages.

In conclusion, our results strongly support that during the efficient cell-wall degradation process and hydrolysis of cellulose in softwood by termites, the native lignin macromolecular assembly undergoes structural modification but with conservation of the abundant *β*-*O*-4' interunit lignin linkage and retention of the original aromatic properties.

We anticipate that further confirmation and elucidation of the absolute structure of this remarkable mechanism will enable the design of enhanced processes for biochemical conversion of lignocellulosic biomass to fuels and chemicals.

## Methods

### Termite cultivation and sample collection

*C. formosanus *termites, collected in Poplarville, Mississippi, were kept at 28°C and 90% humidity, and fed on blocks of Southern pine (*Pinus australis *F. Michx) approximately 17.3 × 3.8 × 1.63 cm in size, The lignin of this wood consists almost exclusively of guaiacyl propane subunits [44]. Termite feces were collected every day and stored at -20°C. For analysis, 1 g each of feces and of the control (the softwood in its natural state with the similar particle size as feces) were freeze-dried and individually ball-milled for 24 hours at room temperature to a fine powder for further analysis. Because the feces were collected from the wood blocks, the possibility of contamination of the feces by minor amounts of undigested wood powder was taken into consideration.

### Solid-state ^13^C CP/MAS NMR analysis

Before investigating the thermal-degradation product of the lignin biopolymer in the control and termite feces, the nature of the polymeric material accumulated in the corresponding cell-wall tissues was initially determined by solid-state NMR spectroscopy studies. The finely ball-milled tissues (~200 mg) of both control and termite-digested softwood materials were individually packed into a 5-mm pencil-type rotor, and the ^13^C CP/MAS spectra were individually recorded using similar acquisition parameters. The solid-state ^13^C CP/MAS analyses (100 MHz) were carried out at NMR Center, Washington State University, using a Bruker Avance 400 spectrometer; (Bruker AXS Inc., Madison, WI, USA), equipped with a double resonance probe (Chemagnetics, Varian, Inc., Palo Alto, CA, USA). For acquisition of ^13^C CP/MAS NMR spectra, a contact time of 0.5 ms, a proton field of approximately 40 kHz during CP and data acquisition, a relaxation delay of 4 seconds and a spinning speed of 5 kHz were used. The spectrum shown is-derived from 18,000 scans, with the chemical shifts given as *δ *ppm.

### Acetyl bromide lignin content analysis

Both the control and termite-digested softwood tissues (1 g each) were individually frozen in liquid nitrogen, ground to powder in a blender (Waring Products, Torrington, CT, USA), with the resulting powders subjected to successive extractions at room temperature for 8 h each with 1:1 toluene-EtOH (100 ml/g)), EtOH (100 ml/g) and H_2_O (100 ml/g), respectively, and then freeze-dried. The resulting extractive-free freeze- dried CWR were ball-milled individually for 2 h to fine powder with a planetary mill (Pulverisette; Fritsch GmbH, Idar-Oberstein, Germany) using agate bowls and balls, and then subjected to acetyl bromide analysis. The lignin contents of extractive-free CWR samples for both the control and termite-digested softwood tissues were estimated by the AcBr method as described previously [30,31].

### Quantitative Py-GC/MS analysis with internal standard

To provide further insight at the structural level into the modifications of the softwood lignin during the termite-digestion process, Py-TMAH-GC/MS analysis was performed. Both control and WFT-digested samples were quickly frozen in liquid nitrogen to halt lignin digestion, and then put directly into a quartz sample tube, with 3,5-dimethoxyphenol added as an internal standard because of its resistance to pyrolysis process [45,46]. Each sample tube contained 1 mg sample and 0.005 mg internal standard. Because the internal standard vaporized in the hot pyrolysis interface during the 3 minute equilibration time and was thus concentrated at the top of the GC column, loss of the internal standard due to thermal fragmentation was avoided. The pyrolysis processes were performed with a pyrolysis autosampler (Model 5000; CDS Analytical, Inc., Oxford, PA, USA) attached to a GC/MS system (Thermo Trace GC 6890N/MSD 5975B; Agilent Technologies, Inc., Bellevue, WA, USA). The samples were first pretreated at 210°C for 3 minutes, then pyrolyzed at a temperature of 610°C for 1 minute, and finally kept in the pyrolysis zone for 56 minutes. The volatile products were separated on a 5% phenyl-methylpolysiloxane non-polar column 30 meters long, with an inner diameter of 0.25 μm, using helium 4.6 as carrier gas (17.3 mL/min), and identified by interpretation of their electron impact (EI) mass spectra and comparison with the NIST MS Search (version 2.0) electronic library. The pyrolysis interface was kept at 210°C and the GC/MS interface at 280°C. The GC/MS was programmed from 40°C (1 minute) to 280°C (15 minutes) at a rate of 6°C/min. The mass spectrometer was operated in EI mode (70 eV) at a source temperature of 230°C. Each analysis was replicated three times using three different pieces of each sample collected at different times.

### Pyrolysis-methylation analysis

Pyrolysis methylation was performed using TMAH as the derivatizing agent for the characterization of lignin in the biomass, which was converted to its corresponding *N*-and *O*-methyl derivatives by TMAH in the pyrolysis chamber, followed by separation and detection by GC/MS. Thermochemolysis reactions of TMAH with lignin in undigested wood and termite fecal samples were carried out as follows. Typically, 1 mg of each sample was placed directly into a quartz sample tube, then covered with 0.5 μL TMAH (25% in methanol). The pyrolysis process was then performed as described above.

## Competing interests

The authors declare that they have no competing interests.

## Authors' contributions

JK performed the termite cultivation, absolute quantification of pyrolysis products analysis and Py-TMAH-GC/MS analysis, and drafted the manuscript. DDL managed the solid-state NMR spectroscopic analysis, helped to analyze other data, and participated in the manuscript editing. DS helped with the experimental design and manuscript editing. SC coordinated the whole study. All authors suggested modifications to the draft, commented on several preliminary versions of the text, and approved the final manuscript.

## References

[B1] Jin Z Katsumata KS Lam TBT Iiyama K Covalent linkages between cellulose and lignin in cell walls of coniferous and nonconiferous woods Biopolymers 2006 83 103 110 10.1002/bip.20533 16673388

[B2] Crawford RL Crawford DL Dizikes GJ Catabolism of the lignin substructure model compound dehydrodivanillin by a lignin-degrading *Streptomyces* Arch Microbiol 1981 129 204 209 10.1007/BF00425251

[B3] Tejado A Peña C Labidi J Echeverria JM Mondragon I Physico-chemical characterization of lignins from different sources for use in phenol-formaldehyde resin synthesis Bioresource Technol 2007 98 1655 1663 10.1016/j.biortech.2006.05.042 16843657

[B4] Bocchini P Galletti GC Camarero S Martinez AT Absolute quantitation of lignin pyrolysis products using an internal standard J Chromatogr A 1999 773 227 232

[B5] Ke J Sun J Nguyen HD Singh D Lee KC Beyenal H Chen S *In-situ *oxygen profiling and lignin modification in guts of wood-feeding termites Insect Sci 2010 17 277 299 10.1111/j.1744-7917.2010.01336.x

[B6] Ke J Singh D Chen S Aromatic compound degradation by the wood-feeding termite *Coptotermes formosanus *(Shiraki) Int Biodeterior Biodegrad 2010 in press

[B7] Geib SM Filley TR Hatcher PG Hoover K Carlson JE Jimenez-Gasco MM Nakagawa-Izumi A Sleighter RL Tien M Lignin degradation in wood-feeding insects P NATL ACAD SCI USA 2008 105 12932 12937 10.1073/pnas.0805257105 PMC2529026 18725643

[B8] Scharf ME Tartar A Termite digestomes as sources for novel lignocellulases Biofuel Bioprod Bopr 2008 2 540 552 10.1002/bbb.107

[B9] Tartar A Wheeler MM Zhou X Coy MR Boucias DG Scharf ME Parallel meta-transcriptome analyses of host and symbiont gene expression in the gut of the termite *Reticulitermes flavipes* Biotechnol Biofuels 2009 2 25 43 10.1186/1754-6834-2-25 19832970 PMC2768689

[B10] Coy MR Salem TZ Denton JS Kovaleva E Liu Z Barber DS Campbell JH Davis DC Buchman GW Boucias DG Scharf ME Phenol-oxidizing laccases from the termite gut Insect Biochem Molec 2010 40 723 732 10.1016/j.ibmb.2010.07.004 20691784

[B11] Maciel GE Davis MF NMR imaging of paramagnetic centers in solids via dynamic nuclear polarization J Magn Reson 1985 64 356 360

[B12] Love GD Snape CE Jarvis MC Determination of the aromatic lignin content in oak wood by quantitative solid state ^13^C-NMR Biopolymers 1992 32 1187 1192 10.1002/bip.360320908

[B13] Singh D. Zeng J Laskar D Lee D Hillcox W Chen S Investigation of wheat straw biodegradation by *Phanerochaete chrysosporium* Biomass Bioenergy 2010 35 1030 1040

[B14] Leary GJ Newman RH Lin SY, Dence CW Cross polarization/magic angle spinning nuclear magnetic resonance (CP/MAS NMR) spectroscopy Methods in Lignin Chemistry 1992 Berlin, Heidelberg: Springer Verlag 146 161

[B15] Schulten H-R Gleixner G Analytical pyrolysis of humic substances and dissolved organic matter in aquatic systems: structure and origin Wat Res 1999 33 2489 2498 10.1016/S0043-1354(98)00493-X

[B16] Geffroy-Rodiera C Grasseta L Sternbergb R Buchc A Amblès A Thermochemolysis in search for organics in extraterrestrial environments J Anal Appl Pyrolysis 2009 85 454 459 10.1016/j.jaap.2008.10.005

[B17] Irwin WJ Analytical Pyrolysis - A Comprehensive Guide 1982 New York: John Wiley & Sons

[B18] Syverud K Leirset I Vaaler D Characterization of carbohydrates in chemical pulps by pyrolysis gas chromatography/mass spectrometry J Anal Appl Pyrolysis 2003 67 381 391 10.1016/S0165-2370(02)00076-1

[B19] Alves A Schwanninger M Pereira H Rodrigues J Analytical pyrolysis as a direct method to determine the lignin content in wood: Part 1: Comparison of pyrolysis lignin with Klason lignin J Anal Appl Pyrolysis 2006 76 209 213 10.1016/j.jaap.2005.11.004

[B20] Rodrigues J Meier D Faix O Pereira H Determination of tree to tree variation in syringyl:guaiacyl ratio of *Eucalyptus globulus *wood lignin by analytical pyrolysis J Anal Appl Pyrolysis 1999 48 121 128 10.1016/S0165-2370(98)00134-X

[B21] Faix O Bremer J Meier D Fortmann I Scheijen MA Boon JJ Characterization of tobacco lignin by analytical pyrolysis and Fourier transform-infrared spectroscopy J Anal Appl Pyrolysis 1992 22 239 259 10.1016/0165-2370(92)85017-F

[B22] Yokoi H Nakase T Ishida Y Ohtani H Tsuge S Sonoda T Ona T Discriminative analysis of *Eucalyptus camaldulensis *grown from seeds of various origins based on lignin components measured by pyrolysis-gas chromatography J Anal Appl Pyrolysis 2001 57 145 152 10.1016/S0165-2370(00)00137-6

[B23] Alves A Rodrigues J Wimmer R Schwanninger M Analytical pyrolysis as a direct method to determine the lignin content in wood: Part 2: Evaluation of the common model and the influence of compression wood J Anal Appl Pyrolysis 2008 81 167 172

[B24] Challinor JM A pyrolysis-derivatisation-gas chromatography technique for the structural elucidation of some synthetic polymers J Anal Appl Pyrolysis 1989 16 323 333 10.1016/0165-2370(89)80015-4

[B25] Clifford DJ Carson DM McKinney DE Bortiatynski JM Hatcher PG A new rapid technique for the characterization of lignin in vascular plants: thermochemolysis with tetramethylammonium hydroxide (TMAH) Org Geochem 1995 23 169 175 10.1016/0146-6380(94)00109-E

[B26] González-Vila FJ Almendros G Del Río JC Martin F Gutiérrez A Romero J Ease of delignification assessment of wood from different Eucalyptus species by pyrolysis (TMAH)-GC/MS and CP/MAS ^13^C-NMR spectrometry J Anal Appl Pyrolysis 1999 49 295 305 10.1016/S0165-2370(98)00097-7

[B27] Kuroda K Nishimura N Izumi A Dimmel DR Pyrolysis of lignin in the presence of tetramethylammonium hydroxide: a convenient method for S/G ratio determination J Agric Food Chem 2002 50 1022 1027 10.1021/jf011198p 11853474

[B28] Kolodziejski W Frye JS Maciel GE Carbon-13 nuclear magnetic resonance spectrometry with cross polarization and magic-angle spinning for analysis of lodgepole pine wood Anal Chem 1982 4 1419 1424

[B29] Eberhardt TL Bernards MA He L Davin LB Wooten LB Lewis NG Lignification in cell suspension cultures of Pinus taeda, *in situ *characterization of a gymnosperm lignin J Biol Chem 1993 268 21088 21096 8407945

[B30] Iiyama K Wallis AFA Determination of lignin in herbaceous plants by an improved acetyl bromide procedure J Sci Food Agric 1990 51 145 161 10.1002/jsfa.2740510202

[B31] Blee K Choi JW O'Connell AP Jupe SC Schuch W Lewis NG Bolwell GP Antisense and sense expression of cDNA coding for CYP73A15, a class II cinnamate-4-hydroxylase, leads to a delayed and reduced production of lignin in tobacco Phytochemistry 2001 57 1159 1166 10.1016/S0031-9422(01)00150-9 11430988

[B32] Iiyama K Wallis AFA An improved acetyl bromide procedure for determining lignin in woods and wood pulps Wood Sci Technol 1988 22 271 280 10.1007/BF00386022

[B33] Mun S Ku C Park S Physicochemical Characterization of pyrolyzates produced from carbonization of lignocellulosic biomass in a batch-type mechanical kiln J Ind Eng Chem 2007 13 127 132

[B34] Lapierre C Jung HG, Buxton DR, Hatfield RD, Ralph J Forage Cell Wall Structure and Digestibility 1993 Madison, WI: American Society of Agronomy, Crop Science Society of America, Soil Science Society of America 133 166

[B35] Van Soest PJ Robertson JB Pigdon WJ, Balch CC, Graham M Standardization of analytical methodology for feeds Systems of analysis for evaluating fibrous feeds 1980 Ottawa, Canada: Int. Dev. Res. Centre 47 60

[B36] Browning BL Methods in Wood Chemistry 1967 New York: A Division of John Wiley & Sons 240

[B37] Martin F Almendrosb G González-Vilaa FJ Verdejoa T Experimental reappraisal of flash pyrolysis and low-temperature thermally assisted hydrolysis and methylation using tetramethylammonium hydroxide for the molecular characterization of humic acids J Anal Appl Pyrolysis 2001 61 133 145 10.1016/S0165-2370(01)00130-9

[B38] Chefetz B van Heemst JDH Chen Y Romaine CP Chorover J Rosario R Guo M Hatcher PG Organic matter transformations during the weathering process of spent mushroom substrate J Environ Qual 2000 29 592 602

[B39] Kuroda K Izumi A Mazumder BB Ohtani Y Sameshima K Characterization of kenaf (*Hibiscus cannabinus*) lignin by pyrolysis-gas chromatography-mass spectrometry in the presence of tetramethylammonium hydroxide J Anal Appl Pyrolysis 2002 64 453 463 10.1016/S0165-2370(02)00047-5

[B40] Filley TR Minard RD Hatcher PG Tetramethylammonium hydroxide (TMAH) thermochemolysis: proposed mechanisms based upon the application of 13C-labeled TMAH to a synthetic model lignin dimmer Org Geochem 1999 30 607 621 10.1016/S0146-6380(99)00040-6

[B41] Challinor JM Characterisation of wood by pyrolysis derivatisation-gas chromatography/mass spectrometry J Anal Appl Pyrolysis 1995 35 93 107 10.1016/0165-2370(95)00903-R

[B42] Lai YZ Sarkanen KV Sarkanen KV, Ludwig CH Isolation and structural studies Lignins: Occurrence, Formation, Structure and Reactions 1971 New York: John Wiley & Sons 165 240

[B43] Filley TR Goodwell B, Nicholas DD, Schultz TP ACS Symposium Serious Wood Deterioration and preservation: Advances in Our Changing World 2003 845 Washington, DC: Am Chem Soc 119 139

[B44] Kim KH Hong J Supercritical CO_2 _pretreatment of lignocellulose enhances enzymatic cellulose hydrolysis Bioresource Technol 2001 77 139 144 10.1016/S0960-8524(00)00147-4 11272020

[B45] Cicerale S Conlan XA Barnett NW Sinclair AJ Keast RSJ Influence of heat on biological activity and concentration of oleocanthal: a natural anti-inflammatory agent in virgin olive oil J Agric Food Chem 2009 57 1326 1330 10.1021/jf803154w 19166297

[B46] Amen-Chen A Pakdel H Roy C Production of monomeric phenols by thermochemical conversion of biomass: a review Bioresource Technol 2001 79 277 299 10.1016/S0960-8524(00)00180-2 11499582

